# Duple-MONDNet: duple deep learning-based mobile net for motor neuron disease identification

**DOI:** 10.55730/1300-0144.5952

**Published:** 2024-08-06

**Authors:** Sony HELEN, Joseph JAWHAR

**Affiliations:** 1 Department of Computer Science Engineering, Anna University, Chennai, Tamil Nadu, India; 2 Department of Electronics and Communication Engineering, Arunachala College of Engineering for Women, Nagercoil, Kanyakumari, India

**Keywords:** Motor neuron disease, Gaussian adaptive bilateral filter, color information feature, local binary pattern, deep learning

## Abstract

**Background/aim:**

Motor neuron disease (MND) is a devastating neuron ailment that affects the motor neurons that regulate muscular voluntary actions. It is a rare disorder that gradually destroys aspects of neurological function. In general, MND arises as a result of a combination of natural, behavioral, and genetic influences. However, early detection of MND is a challenging task and manual identification is time-consuming. To overcome this, a novel deep learning-based duple feature extraction framework is proposed for the early detection of MND.

**Materials and methods:**

Diffusion tensor imaging tractography (DTI) images were initially analyzed for color and textural features using dual feature extraction. Local binary pattern (LBP)-based methods were used to extract textural data from images by examining nearby pixel values. A color information feature was then added to the LBP-based feature during the classification phase for extracting color features. A flattened image was then fed into the MONDNet for classifying normal and abnormal cases of MND based on color and texture features.

**Results:**

The proposed deep MONDNet is suitable because it achieved a detection rate of 99.66% and can identify MND in its early stages.

**Conclusion:**

The proposed mobile net model achieved an overall F1 score of 13.26%, 6.15%, 5.56%, and 5.96% compared to the BPNN, CNN, SVM-RFE, and MLP algorithms, respectively.

## 1. Introduction

Motor neuron disease (MND) constitutes a group of debilitating neurodegenerative diseases harming the neural units that govern voluntary movements of the muscles [1,2]. These diseases, which include amyotrophic lateral sclerosis (ALS), primary lateral sclerosis (PLS), and progressive muscular atrophy (PMA), are characterized by the gradual degeneration of motor neurons, leading to muscle weakness, atrophy, and eventually paralysis [3,4]. Early diagnosis and effective management of MND are crucial for improving the quality of life of affected individuals, but this is often significantly challenging due to the complexity and heterogeneity of these conditions [5,6].

Deep learning has been an effective method for illness categorization and the analysis of medical images over the past decade. Convolutional neural networks (CNNs) [7] and recurrent neural network (RNNs) in particular have demonstrated excellent ability in extracting key characteristics from clinical records, electrodiagnostic data, and clinical imagery [8]. These models aid in the early detection and accurate classification of MND by analyzing various data sources, such as electromyography (EMG) signals, magnetic resonance imaging (MRI) scans [9], and patient clinical histories [10]. Leveraging deep learning for MND classification not only holds the potential to streamline the diagnostic process but may also make it possible to identify subtle patterns and biomarkers that might otherwise go unnoticed by human clinicians [11].

One of the key advantages of deep learning [12] in MND classification is its ability to learn from vast datasets. By training on diverse and extensive datasets containing information from patients with various disease stages and demographics, deep learning models generalize their knowledge, enhancing their diagnostic accuracy [13]. Additionally, deep learning is not limited to a single data type; it integrates information from multiple sources such as genetic profiles and clinical notes to provide a comprehensive understanding of the disease. This multimodal approach [14] holds promise for a more holistic assessment of MND [15]. The average lifespan of someone with MND is 2 to 3 years after diagnosis, though individual circumstances may change this. Many years may pass after a diagnosis for some people. The early stage of MND is very difficult to diagnosis. Artificial intelligence (AI) has proliferated in recent years across all scientific disciplines [16]. The early detection of MND is now more accurate and precise thanks to the application of AI in medicine. However, early detection of MND remains a challenging task and manual identification is time-consuming. To overcome these challenges, a novel deep learning-based duple-MONDNet model is proposed in this study for identifying healthy individuals and patients affected by MND while also employing advanced deep learning models such as CNNs, long short-term memory (LSTM), and the you-only-look-once (YOLO) algorithm [17] to contribute to the detection of MND. The key contributions of the proposed duple-MONDNet model are as follows:

- Initially, diffusion tensor imaging (DTI) tractography images are used in the duple feature extraction phase for extracting the color and textural features of the images.
- A local binary pattern (LBP)-based method extracts the textural data of an image by considering the neighboring pixel values.
- The color information feature (CIF) is then added with the LBP-based feature for color feature extraction in the classification phase.
- Afterward, the extracted color and texture features of the images are flattened and given as input to the mobile net for classifying MND.
- Finally, the mobile net is employed for classifying healthy cases and abnormal cases of MND.

Relevant studies are summarized in detail in Section 2, the proposed duple-MONDNet for detecting MND is explained in detail in Section 3, and the experimental findings and a discussion are presented in Section 4. The study is concluded in Section 5, which addresses possibilities for future research.

## 2. Literature survey

Numerous researchers have used digital image processing and classification techniques to conduct studies on recognizing both healthy cases and abnormal cases of MND. Diverse studies have been published about recent developments in deep learning and machine learning techniques.

In 2019, Agosta et al. [18] studied a significant cohort of people with MND and explored the prognostic effect of multimodal brain MRI for survival. Multivariable medical and mental features were used to build the Royston–Parmar survival model. The integrated clinical and MRI model with specific front-temporal gray matter densities and mobility vector MRI parameters achieved an AUC value of 0.89.

In 2019, Lauraitis et al. [19] proposed a smartphone app for automated decision aid for cognitive task-based assessments of motor diseases of the neuron system. A backpropagation neural network (BPNN) classifier was utilized to examine the data and provide results. The rate of success in identifying early prodromal symptoms of motor illnesses was 86.4%. However, the proposed method showed a low reliability rate compared to other models.

In 2019, Hassanpour et al. [20] proposed a multiclass motor imaging electroencephalogram signal classification end-to-end deep neural network. Deep Belief Networks (DBNs) and the Stacked Sparse Autoencoder (SSAE), as two generative deep learning (GDL) frameworks, were used in an end-to-end fashion. Additionally, the effectiveness of the suggested methodology was assessed both with and without the class-specific features (CS) and nonrecurrent (NR) phases. For the DBN framework, the suggested method attained reliability of 91.54% and 90.21%, accordingly.

In 2020, Ramakrishnan et al. [21] designed a cross-power spectral density-based wheelchair control system for the detection of MND. A CNN model was employed for the detection of MND using patients’ eye movements. Qualified users achieved total reliability of 93.51% in the evaluation but the suggested method obtained a lower accuracy rate compared to other existing methods.

In 2020, Zhang et al. [22] designed a hybrid neural network to enhance the detection of motor imagery signals. To enhance the ability to classify motor functions, a generative adversarial network was presented. Short-time Fourier transform (STFT) was used to convert the time sequence data into spectrogram visuals. The hybrid deep convolutional generative adversarial network (DCGAN) fared better than previous categorizations and achieved reliability with average kappa scores of 0.564 and 0.677 from dataset. However, the obtained accuracy level was still not sufficient.

In 2021, Greco et al. [23] suggested utilizing only blood data to identify and classify patients with upper and lower MND. For categorizing each patient into either the ALS class or lower MND class, a support vector machine with recursive feature elimination (SVM-RFE) was implemented. The experiment yielded an accuracy rate of 94% for the classification, and this was lower than the rates of other approaches.

In 2021, Subasi and Mian Qaisar [24] suggested a powerful combination of multiscale principal component analysis (MSPCA) and ensemble learning-based algorithms for the classification of MND. Employing ECG signals, a wavelet transform based on the Daubechies method was used to achieve noise reduction. The ensemble learning method had accuracy values of 98.69% and 94.83%.

In 2022, Sekar et al. [25] proposed a neural machine learning model to recognize MND and forecast its effects on health. Based on both historical data and current knowledge, the machine learning system was used to forecast the effects of MND. The authors reported rates of 93.28% for bulbar palsy, 91.44% for tendon erosion, and 93.22% for polytopic paralysis. The experiment attained a low reliability rate.

In 2022, Bede et al. [26] presented phenotypic classification of MND patients using radiological disease load variations. Applying the multilayer perceptron (MLP) approach, the rate of classification for ALS was 93.7%, while a poor diagnostic accuracy rate was found for PLS at 43.8%. The experiment yielded a low level of success for the classification of illnesses.

In 2023, Toh et al. [27] used spinal and brain MRI measurements in a single region to directly evaluate the neurodegeneration in MND. A total of 75 MND patients and 13 normal controls underwent MRI. Utilizing FreeSurfer software, volumetric T1-weighted images were used to quantify the precentral gyral width. The experiment achieved success of 95% but the reliability level was not high enough for detection.

As seen in the studies described above, MND has been detected utilizing various techniques. To determine and categorize diseases, researchers employ approaches like the preliminary processing of images and categorize diseases using various training models. However, the techniques utilized to date provide low reliability rates compared to advanced deep learning approaches. To overcome this, a novel duple-MONDNet model is proposed in this study for the early detection of MND.

## 3. Proposed method

**Figure 1:**
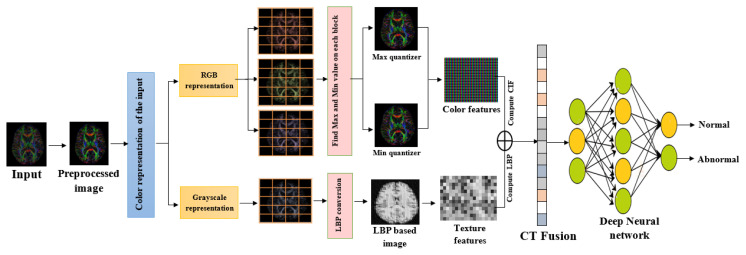
Schematic illustration of the proposed duple-MONDNet model.

A novel deep learning-based duple-MONDNet model was designed for identifying healthy individuals and patients affected by MND. Initially, images were fed into the dual feature extraction phase to extract color and textural features. The LBP-based operator then extracted the textural data of images by considering neighboring pixel values. The CIF was then added with the LBP-based feature for color feature extraction in the classification phase. Afterward, the extracted color and texture features of images were flattened and given as input to the mobile net for classifying normal and abnormal cases. Finally, the mobile net was employed for classifying the early stages of MND. Figure 1 depicts the proposed duple-MONDNet model.

### 3.1. Dataset description

In this section, the compiled dataset and the data augmentation process are described for the enhancement of images in the dataset and detection of MND. The images were collected from the Pranav Diagnostics Centre in Nagercoil, Tamil Nadu, India. The dataset comprised 78 normal cases and 52 abnormal images. To enhance the dataset, the collected images underwent augmentation. The study’s experimental setup was prepared using Spyder, an Anaconda navigator, running on a PC equipped with Windows 10 OS. The PC had an Intel i5 core processor with clock speed of 2.10 GHz and a 16GB RAM system. Additionally, the performance of the proposed model was evaluated with several other deep learning models.

Table 1 displays the distribution of disease classes in the dataset before and after augmentation. Initially, the dataset consisted of 130 total images sourced from our self-prepared dataset, with 78 representing normal brain scans and 52 representing abnormal brain scans. Following augmentation, the dataset underwent significant expansion, with the total number of images increasing to 3120 for the self-prepared dataset. Notably, the augmentation process substantially increased the number of normal images from the self-prepared dataset to 1872, while the number of abnormal images from the self-prepared dataset similarly rose to 1248. As a result, the augmented dataset comprised a total of 3250 images, constituting a larger and more balanced dataset for training and analysis purposes.

### 3.2. Gaussian adaptive bilateral (GAB) filter

A GAB filter was used in the preprocessing stage to reduce the distortion in the input images. The principle of bilateral filtering is combined with adaptive parameter adjustments in GAB filters, which effectively denoise medical images. This is crucial for the identification of motor illnesses by deep learning since efficient data maintenance is required for reliable diagnosis and evaluation. The proposed method significantly enhanced the quality of the images. The bilateral filter and input image *I_p_* and guidance *G_d_* are different, as shown in Eq. (1):


(1) $$f(v)=\Sigma_{u}\;\left(W_{v,u}^{G_{d}}\right)\;(G_{d})I_{u}$$

Here, *I_u_* represents the source image and $W_{v,u}^{G_{d}}$ is given in Eq. (2):


(2) $$W_{v,u}^{G_{d}}=\frac{1}{Nor_{f}}\;\text{exp }\left[-{\left\|\frac{v-u}{-\sigma_{z}^{2}}\right\|}^{2}\right]$$

From Eq. (2), *Nor_f_* denotes the normalizing factor and the Gaussian spatial filter is depicted by $\;\text{exp }[-{\left\|\frac{v-u}{-\sigma_{z}^{2}}\right\|}^{2}]$. The GAB kernel is expressed in Eq. (3):


(3) $${ℌ}_{v,u}^{gab}(I,G^{-})=\frac{1}{Nor_{f}}\;\text{exp }[-{\left\|\frac{v-u}{-\sigma_{z}^{2}}\right\|}^{2}]exp[-{\left\|\frac{I_{v}-G_{d}^{-}}{-\sigma_{s}^{2}}\right\|}^{2}]$$

Here, $-\sigma_{z}^{2}$ represents the difference in intensities. *G_d_* ^−^ is obtained from Eq. (1) and (3) and $\text{exp }\left[-{\left\|\frac{I_{v}-G_{d}^{-}}{-\sigma_{s}^{2}}\right\|}^{2}\right]$ is the range kernel.


(4) $$f(v)=\sum_{v}\left(W_{v,u}^{G_{d}}\right)\;[I_{p},{G_{d}}^{-}]I_{u}$$

Final output *f*(*v*) of the GAB filter is defined in Eq. (4). Noise-free images are used as input to the CT fusion to extract the key characteristics for categorizing the DTI into normal cases and abnormal cases of MND.

### 3.3. Proposed duple-MONDNet

A deep MONDNet is proposed in this study for detecting MND in its earliest stages using DTI images from the compiled datasets. The CIF and LBP were used for color and texture feature extraction and the mobile net was used for classifying normal cases and abnormal cases of MND.

#### 3.3.1. Color information feature (CIF) block

The CIF block is a specialized component in deep learning models designed for disease detection from medical images, particularly where color information plays a critical role. This block is engineered to efficiently extract relevant color-related features from input images, enhancing a model’s ability to discriminate between healthy and diseased tissues or structures. The CIF block is designed to capture intricate color patterns and variations within medical images. It typically consists of a series of convolutional layers, each with learnable filters that convolve over the input image’s color channels (e.g., RGB or other color representations). These filters are designed to detect specific color gradients, textures, or patterns indicative of disease-related characteristics. The output of these convolutional layers is then processed to generate color-related features.

In addition to its convolutional layers, the CIF block may also incorporate advanced techniques like attention mechanisms or feature fusion. These enhancements enable a model to prioritize certain color-related features or integrate them with other relevant information extracted from images, further improving diagnostic accuracy.

**Figure 2:**
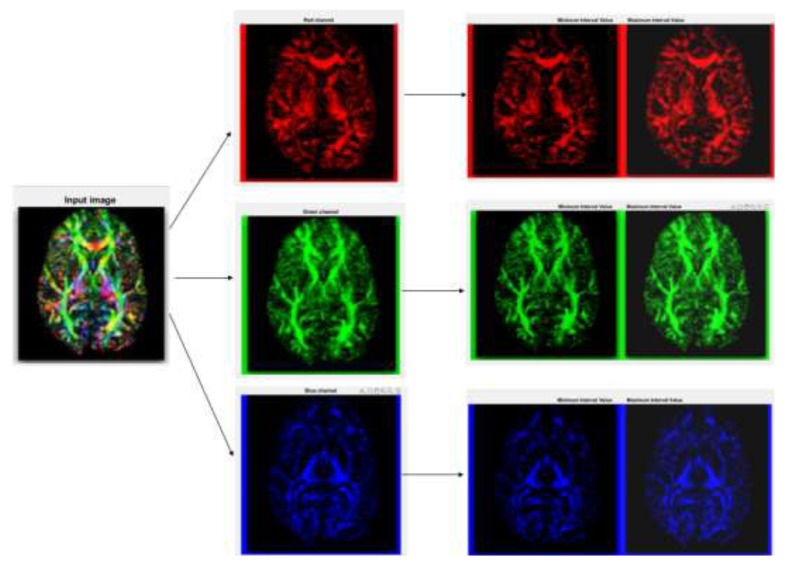
Schematic RGB representation of min and max quantizer identification.

The CIF block stores details about color, including pixel value, contrast, and color dispersion. A color image must initially be divided into many image chunks in the first stage. Figure 2 depicts RGB conversion in a CIF block.

Utilizing CIF blocks, the input image undergoes a transformation whereby intricate color patterns are extracted and segregated into distinct channels of red, green, and blue. Each channel is then subjected to min and max quantizer identification, which aids in increasing the features essential for accurate disease detection. The CIF block captures intricate color patterns and variations within medical images. These patterns often correspond to disease-related characteristics. By identifying these patterns, the CIF block detects signs of disease that are missed by other methods. The CIF block is robust to variations in lighting and color due to its focus on relative color information. This makes it more reliable in different imaging conditions. By prioritizing certain color-related features and integrating them with other relevant information extracted from the image, the CIF block improves the diagnostic accuracy. This is particularly important in medical imaging, where accurate diagnosis can significantly impact patient outcomes. The CIF block processes images in chunks, which is more computationally efficient. This is crucial in medical imaging, where large volumes of data need to be processed quickly. The CIF block enhances the precision of disease detection in medical images by efficiently extracting and processing color-related features. This not only improves the model’s ability to distinguish between healthy and diseased tissues but also captures intricate color patterns and variations within the images, which are often indicative of disease manifestations.

The color reduction method for the color quantizers was applied in this study after obtaining the balanced tree. Each color quantizer, such as min and max quantizers, receives a single value representation as a result of the color reduction process. Let $T_{min}=\left\{\hat{s_{1}},\hat{s_{2}},\ldots ,{\hat{s}}_{k_{min}}\right\}$ and $T_{max}=\{\hat{s_{1}},\hat{s_{2}},\ldots ,{\hat{s}}_{k_{max}}\}$ be the set of input images from the minimum and maximum quantizer, respectively. Here, *k_min_* and *k_max_* are the dimensions of the minimum and maximum colors. *s*_min_ (*u,v*) and *s*_max_ (*u,v*) are the minimum and maximum quantizers on image block (*u, v*). The color extraction method for the minimum quantizer is shown in Eq. (5):


(5) $$\xi \{s_{min}\}={\hat{s}}_{c}$$

Here, c= {1,2, …, *s_min_*}and *ξ*{.} signifies the color extraction process for the input images. The color extraction process for the max quantizer is shown in Eq. (6):


(6) $$\xi \{s_{max}\}={\hat{s}}_{d}$$

Here, c= {1,2, …, *s_max_*} and the above equation demonstrates the nearest pair for the maximum quantizer. The feature extraction phases for *CIF_min_* and *CIF_max_* are derived by utilizing Eq. (7) and (8):


(7) $${CIF}_{min}(a)=\chi [\xi (s_{min}(u,v))\}={\hat{s}}_{c}|J=1,2,\ldots ,\frac{l}{\ell };J=1,2,\ldots ,\frac{N}{K}]$$

(8) $${CIF}_{max}(a)=\chi [\xi (s_{max}(u,v))\}={\hat{s}}_{d}|J=1,2,\ldots ,\frac{l}{\ell };J=1,2,\ldots ,\frac{N}{K}]$$

*χ*(.) signifies the probability factor of the min and max quantizers. Here, *c*= 1,2, …, *s_min_* and *d*= 1,2, …, *s_max_* are identical widths in color extraction. The processing of several quantizers such as minimum and maximum quantizers from a color image leads to the creation of a CIF block comprising a set of 1 × 1 pointwise convolutions (PWConv), a channel shuffling operation, a set of 3 × 3 depthwise separable convolutions (DWConv), and a channel reordering action. The feature map’s result is demonstrated in Eq. (9):


(9) $$s_{f}(ℒ,K)=(Q\times N)(ℒ,K)=\Sigma_{a}\Sigma_{b}Q(a,b)\;Q\;(ℒ-a,K-b)$$

Here, (ℒ, 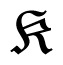) shows the input and the kernel of the currently accessed phase and *s_f_* represents the characteristic image. The extracted features of the CIF block are fused with the LBP block for feature extraction in the classification phase.

#### 3.3.2. Local binary pattern (LBP) block

LBP is a texture descriptor frequently used in conjunction with deep learning techniques for various computer vision tasks. Its primary role in deep learning is to serve as a feature extraction method. While working with deep neural networks, and particularly CNNs, LBP is applied as a preprocessing step to capture essential texture information from images. By extracting LBP-based features, the network focuses on learning more complex and discriminative features during training.

By extracting LBP features from unlabeled or partially labeled data and using them as input for a deep learning model, it is possible to perform unsupervised or semisupervised feature learning, which is especially beneficial in medical imaging or other domains with scarce annotated data. LBP plays a valuable role in deep learning by providing a texture-based feature extraction method that enhances the capabilities of deep neural networks in various computer vision applications, especially when data are limited or when capturing local texture information is critical for accurate predictions. The process starts with a grayscale brain scan image. Grayscale is used to simplify the image while retaining essential information. Color images are converted to grayscale, where each pixel corresponds to the intensity of the light that it represents. LBP is a very efficient texture operator that labels the pixels of an image by thresholding the neighborhood of each pixel and considers the result as a binary number. It is robust against monotonic grayscale changes and has shown excellent results in detecting MND. The LBP operation transforms grayscale images into texture maps. These maps emphasize the different textures present in the images, which correspond to different tissue types in the brain. A texture map makes certain features more distinguishable than the original grayscale image. This method is useful in medical imaging to extract specific features from images for further analyzing brain diseases. The enhanced contrast image provided by LBP-based feature extraction aids in identifying areas of interest that might be less noticeable in the original grayscale image.

**Figure 3:**
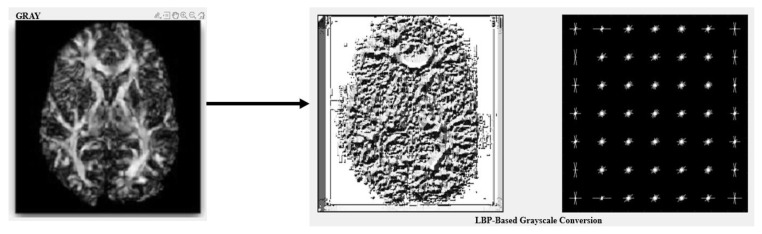
Diagrammatic representation of LBP-based feature extraction.

Diagrammatic representations of LBP-based feature extraction are shown in Figure 3. The textural characteristics of images are typically captured by image processing systems using LBP and its variants. For retrieval, the visual representation of the LBP pattern serves as an attribute classifier. Before LBP is calculated, a color image is first converted to grayscale. By evaluating the contents of the central pixel compared to those of its peers, the LBP generates its code by taking into account the characteristics of the surrounding pixels. The local ternary pattern is a variant of the LBP function, generating three distinct areas in the gray values of the primary pixel and adjacent pixels. The LBP-based feature extraction phase of input image *I* of size *M* × *N* in RGB color space is initially transformed into the interband average depiction as in Eq. (10):


(10) $$P(x,y)=\frac{1}{3}[R_{f}(x,y)+G_{y}(s,m)+B_{y}(s,m)]$$

Here, *x* = 1,2, … ,*M* and *y* = 1,2, …, *N.* The factor ( 
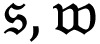
) represents the pixel location of an image. R, G, and B signify red, green, and blue color space. For image *z_i_* , the *q*th convolutional layer’s characteristic map can be given as *conv_i_z_i_* = [*conv_i_* 1 *z_i_*, *conv_i_* 2 *z_i_*, …, *conv_i_z_i_ f z_i_*], where f is the quantity of filters in the *q*th layer of the proposed model. For each pixel (*u*, *v*) in *conv_i_* 1 *z_i_*, the LBP block is computed as in Eq. (11):


(11) $$LBP_{s,r}(u,v)=\sum_{\alpha =1}^{s}\varrho (t_{v}-t_{u})\times 2^{n-1}$$

In the above equation, *u* ≥ 0 *t_v_* and tu(*α* = ···*p*) denote the intensity values of pixel (*u*,*v)* , and p is the neighboring pixel. To express information about the texture of the image, the occurrences of various binary patterns are gathered into a histogram.

Let $B_{h_{z_{i}}^{qf}}$ be the texture of the histogram of extracted features from $h_{z_{i}}^{qf}$. For image *z_i_*, the texture histogram is shown in Eq. (12):


(12) $$B(z_{i})=\{B_{h_{z_{i}}^{1}}.B_{h_{z_{i}}^{2}},\ldots ..,B_{h_{z_{i}}^{Q}}\}$$

Here, *B*(*z_i_*) is the convolutional layer in the fine-tuned model. $B\left(z_{i}^{RGB}\right)$ and $B\left(z_{i}^{gray\;scale}\right)$ are the color texture histograms from the models. The gray-level value of pixel (*t_v_*,*t_u_*) in the grayscale factor of the adjacent pixels is denoted in Eq. (13):


(13) $$s(x)=\{\begin{array}{ll} 1 & if\;x\geq 0\\ 0 & if\;x\geq 0 \end{array}$$

In deep learning for MND classification, the fusion of color and texture features from DTI images can be achieved using advanced neural network frameworks and techniques. The fusion of color and texture features in medical imaging, such as DTI scans, is a valuable approach for MND classification. This fusion strategy enhances the sensitivity and specificity of classification models, allowing for more accurate and robust diagnoses of MND cases. Deep learning techniques such as duple-MONDNet can be employed effectively to integrate color and texture information from DTI scans and improve the overall performance of disease classification systems. By combining color and texture information in a deep learning model, the model becomes more adept at discriminating subtle pathological patterns, leading to more precise and reliable diagnoses of MND from DTI scans.

#### 3.3.3. Mobile net

The mobile net is made up of convolutional structures with depthwise separable convolutions that are more computationally efficient than regular convolutions. The network starts with a series of standard convolutional layers to capture low-level features, followed by depthwise separable convolutions that efficiently extract spatial information while reducing computational load. Depthwise separable convolutions are followed by pointwise convolutions that combine features from different channels. Batch standardization and the ReLU layer are applied to enhance network training and stability. The mobile net typically ends with a global average pooling layer and a fully connected SoftMax layer for categorization.

Following each convolution process, the batch normalization procedure and the ReLU activation feature are employed to achieve automatic data distribution correction. Deep and separable convolution networks speed up mobile net training and significantly reduce the cost. The standard convolution structure is denoted in Eq. (14):


(14) $${ℜ}_{r}=\Sigma_{k}\;\omega_{k,l}\cdot {\text{I}}_{k}$$

Here, *k* and *l* are the input and output phases. *ω_k,l_* is the kernel, while I*_k_* signifies the given data and feature attribute, utilizing the style of minimum padding. If the dimension of given input data I*_k_* is *H_i_*_*_p_*_ × *H_i_*_*_p_*_, the *l* kernel and *k* channel phase are present before the feature map is accessed. The computing cost of the standard convolutional layer is shown in Eq. (15):


(15) $${ℒ}_{r}={\text{H}}_{o/p}\times {\text{H}}_{o/p}\times k\times l\times {\text{H}}_{i_{p}}\times {\text{H}}_{i_{p}}$$

The depthwise convolutional phase is demonstrated in Eq. (16), where ℘_1_*_,k_* represents the kernels and I*_k_* denotes input data:


(16) $$\chi_{k}=\Sigma \;{℘}_{1,k}\cdot {\text{I}}_{k}$$

In depthwise convolution, *k* filters with *l* channels and H*_o_*_/_*_p_* × H*_o_*_/_*_p_* length are provided. It is important to have *l* filters during the pointwise iteration with 1 × 1 dimensions using *k* channels. The computational cost of the extensive separable convolution structure is calculated utilizing Eq. (17):


(17) $${ℱ}_{\nu }={\text{H}}_{o/p}\times {\text{H}}_{o/p}\times k\times {\text{H}}_{i_{p}}\times {\text{H}}_{i_{p}}+k\times l\times {\text{H}}_{i_{p}}\times {\text{H}}_{i_{p}}$$

**Figure 4:**
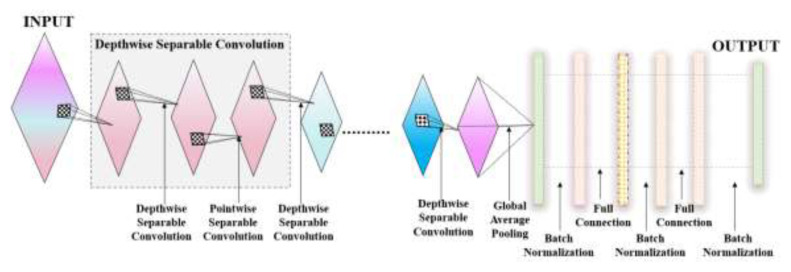
Architecture of the proposed model.

Considering the above equation for computational cost together with the standard convolutional method, the cost of the proposed approach is reduced by $\frac{1}{l}+\frac{1}{{{\text{H}}^{2}}_{o/p}}$. The features after color extraction are fed into the mobile net for classification of MND cases. The architecture of the proposed mobile net is shown in Figure 4.

By using deep and separable convolutional structures, the mobile net allows for rapid training and reduced calculations. It can accordingly be employed for classifying normal and abnormal cases.

## 4. Results and discussion

**Figure 5:**
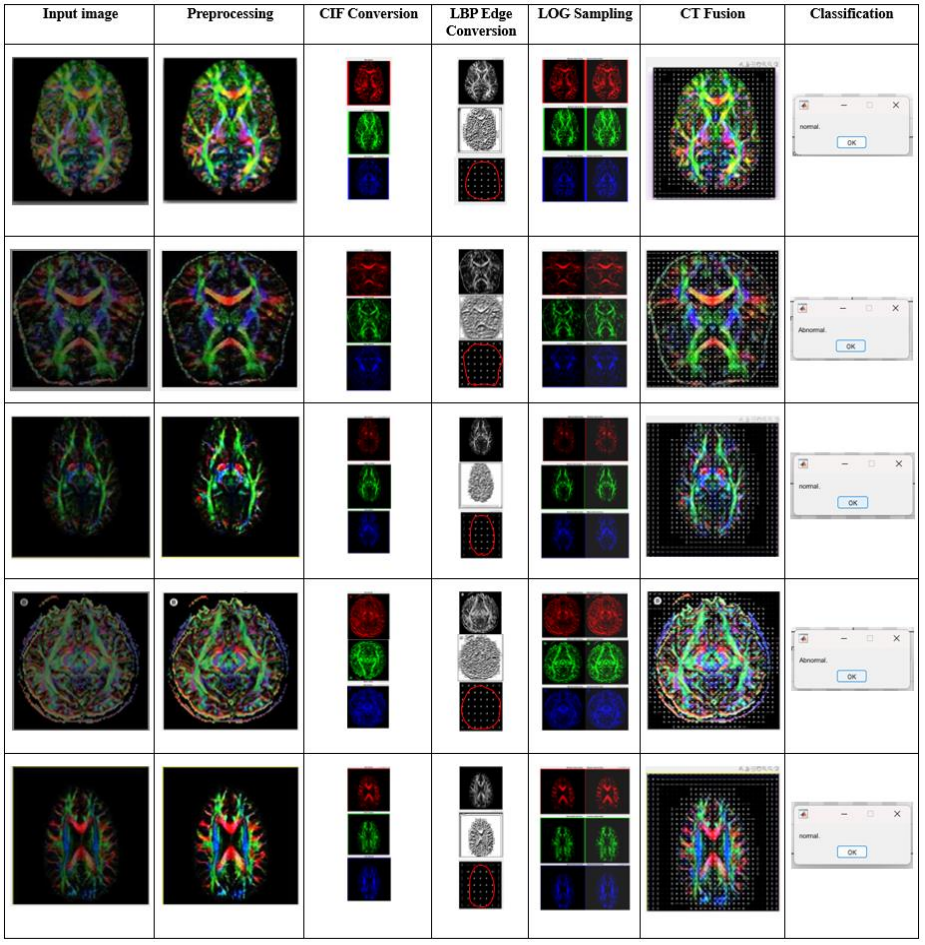
Experimental outcomes of the proposed duple-MONDNet model.

Cases of MND were categorized by utilizing the compiled dataset. Figure 5 shows the experimental outcome of the proposed duple-MONDNet model utilizing that dataset. The input images (column 1) were preprocessed using a GAB filter (column 2) to reduce distortion and improve the quality of the input images. Concurrently, these preprocessed images were fed to the color conversion block (column 3) for color feature extraction. The CIF block was combined with the LBP block (column 4) for color and texture feature extraction of images in the classification phase. The minimum and maximum values of the RGB conversion images and the extracted features are shown in columns 5 and 6 of Figure 5, respectively. Finally, the mobile net was employed for classifying normal cases and abnormal cases of MND (column 7).

### 4.1. Performance analysis

The results of this study include data on specific variables in MND recognition, including precision, sensitivity, specificity, accuracy, recall, and F1 score. Basic variables including true positive (*T_u_P_v_*^+^), true negative (*T_u_N_v_*^+^), false positive (*f_l_P_v_^+^* ), and false negative (*f_l_N_v_*^+^) rates were used as evaluation metrics. Accuracy in MND classification reflects the percentage of correctly classified instances, providing a straightforward measure of overall model performance. Using Eq. (18), the accuracy was evaluated:


(18) $$A=\frac{T_{u}{P_{v}}^{+}+T_{u}{N_{v}}^{+}}{T_{u}{P_{v}}^{+}+T_{u}{N_{v}}^{+}+f_{l}{P_{v}}^{+}+f_{ul}{N_{v}}^{+}}\times 100$$

Precision is a crucial performance metric in deep learning for motor disease classification. It measures the accuracy of positive predictions among all predicted positive cases:


(19) $$P=\frac{T_{u}{P_{v}}^{+}}{T_{u}{P_{v}}^{+}+f_{l}{P_{v}}^{+}}$$

(20) $$Re=\frac{T_{u}{P_{v}}^{+}}{T_{u}{P_{v}}^{+}+f_{l}{P_{v}}^{+}}$$

(21) $$F1-Score=\frac{2PreRec}{Pre+Rec}$$

Here, *T_u_P_v_*^+^ and *T_u_N_v_*^+^ signify the true positives and negatives of the input images, while *f_l_P_v_*^+^ and *f_l_N_v_*^+^ represent the false positives and negatives of the images.

The effectiveness of the proposed duple-MONDNet model in classifying the early stages of MND together with normal cases is shown in Table 2. The proposed model yielded an accuracy rate of 99.66% and an F1 score of 98.44%.

**Figure 6:**
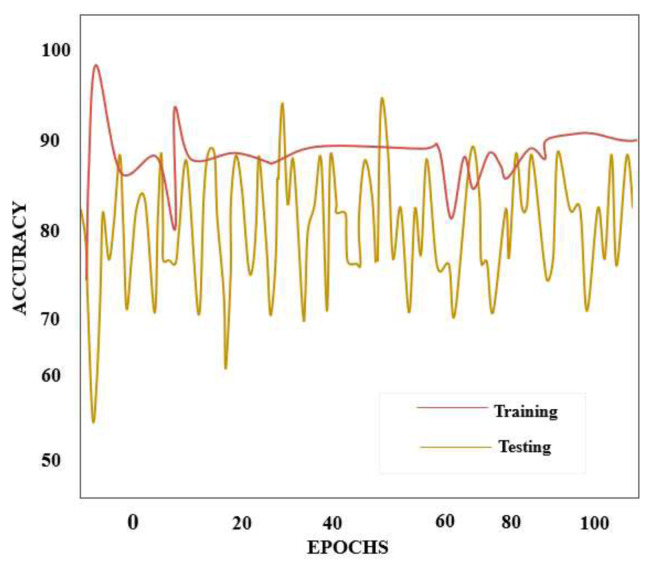
Accuracy curve of the proposed duple-MONDNet model.

**Figure 7:**
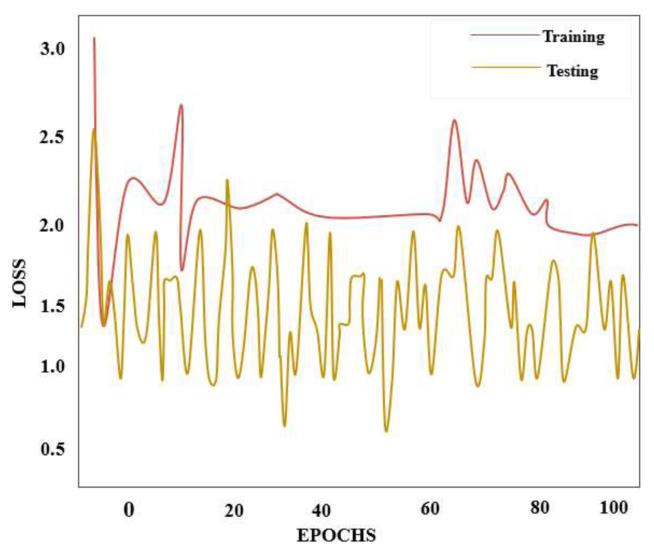
Loss curve of the proposed duple-MONDNet model.

The accuracy value is displayed on the vertical axis of the reliability curve in Figure 6, while the quantity of phases is plotted on the horizontal axis. The epoch and deficit scale in Figure 7 shows that data loss is minimized for the duple-MONDNet model as the epochs are increased.

The proposed duple-MONDNet model classifies the early stages of MND using DTI images. The number of training epochs was deemed sufficient for attaining the best testing accuracy. With 100 epochs, the proposed model attained 99.66% testing accuracy with a low percentage of errors.

### 4.2. Comparative analysis

The effectiveness of each considered neural network was assessed to verify that the duple-MONDNet model had higher accuracy. ResNet, AlexNet, and GoogleNet, as neural network classifiers, were assessed for performance together with the proposed duple-MONDNet model. Quality was estimated using various measures including accuracy, specificity, and recall, which are superior to those employed by conventional deep learning networks.

Table 3 compares the maximal capacity for categorization over many common deep learning connections. The conventional deep learning networks failed to produce more significant results than the proposed duple-MONDNet. The proposed mobile net raised the overall F1 score by 2.59%, 3.51%, and 4.14%, respectively.

To allow the assessment of the effectiveness of different strategies, Table 4 provides the experimental results of test images from the compiled dataset. One measure of performance for evaluating prior models was the efficiency of categorization. Comparing the proposed model to the BPNN, CNN, SVM-RFE, and MLP approaches resulted in F1 scores of 13.26%, 6.15%, 5.56%, and 5.96%, respectively. The older networks did not achieve superior results compared to the proposed duple-MONDNet model. The duple-MONDNet model seems to be quite reliable for distinguishing between normal and abnormal cases.

## 5. Conclusion

In this study, novel deep learning-based duple feature extraction was proposed for the early detection of MND. DTI images were initially analyzed for color and textural features by using dual feature extraction. LBP-based methods were used to extract textural data from an image by examining nearby pixel values. The CIF block was then added to the LBP-based feature during the classification phase for extracting color features. A flattened image was then fed into the mobile net as a classifier that uses the color and texture features of images to categorize normal cases and abnormal MND cases. MND cases were detected with average classification accuracy of 99.66%. The proposed model achieved overall F1 scores of 13.26%, 6.15%, 5.56%, and 5.96% compared to the BPNN, CNN, SVM-RFE, and MLP approaches. In the future, the proposed model will be extended with advanced deep learning techniques. An advanced color extraction model could also be implemented for improving the diagnosis rate.

## Figures and Tables

**Table 1:** Description of the dataset for the proposed model.

Disease classes	Before augmentation	After augmentation	Total images
	Self-prepared dataset	Self-prepared dataset	
Normal	78	1872	1950
Abnormal	52	1248	1300
Total	130	3120	3250

**Table 2:** Evaluation outcomes of the proposed duple-MONDNet model.

Classes	Accuracy	Precision	Recall	Specificity	F1 score
Normal	99.64	98.09	98.33	97.55	98.67
Abnormal	99.68	97.14	97.69	96.66	98.22

**Table 3:** Comparisons with several traditional networks.

Networks	Accuracy	Precision	Recall	Specificity	F1 score
ResNet [28]	97.35	97.27	96.36	95.44	97.07
AlexNet [29]	96.17	95.98	97.30	97.453	96.15
GoogleNET [30]	95.18	94.34	95.63	95.11	95.52
Proposed model	98.99	98.19	97.21	97.82	98.74

**Table 4:** Comparisons of accuracy between existing methods and the proposed duple-MONDNet model.

Reference	Method	Accuracy
Lauraitis et al. [19]	BPNN	86.4%
Ramakrishnan et al. [21]	CNN	93.51%
Greco et al. [23]	SVM-RFE	94%
Bede et al. [26]	MLP	93.7%
Proposed model	Duple-MONDNet	99.66%

## References

[1] Leigh PN Ray-Chaudhuri K Motor neuron disease Journal of Neurology, Neurosurgery, and Psychiatry 1994 57 8 886 10.1136/jnnp.57.8.886 8057109 PMC1073069

[2] Tiryaki E Horak HA ALS and other motor neuron diseases Continuum: Lifelong Learning in Neurology 2014 20 5 1185 1207 10.1212/01.CON.0000455886.14298.a4 25299277

[3] Talbot K Motor neuron disease Practical Neurology 2009 9 5 303 309 10.1136/jnnp.2009.188151 19762894

[4] Puls I Jonnakuty C LaMonte BH Holzbaur ELF Tokito M Mutant dynactin in motor neuron disease Nature Genetics 2003 33 4 455 456 10.1038/ng1123 12627231

[5] Lev S Halevy DB Peretti D Dahan N The VAP protein family: from cellular functions to motor neuron disease Trends in Cell Biology 2008 18 6 282 290 10.1016/j.tcb.2008.03.006 18468439

[6] Chiò A Meineri P Tribolo A Schiffer D Risk factors in motor neuron disease: a case-control study Neuroepidemiology 1991 10 4 174 184 10.1159/000110267 1745327

[7] Sivasankari B Shunmugathammal M Appathurai A Kavitha M High-throughput and power-efficient convolutional neural network using one-pass processing elements Journal of Circuits, Systems and Computers 2022 31 13 2250226 10.1142/S0218126622502267

[8] David AS Gillham RA Neuropsychological study of motor neuron disease Psychosomatics 1986 27 6 441 445 10.1016/S0033-3182(86)72673-X 3737834

[9] Sundarasekar R Appathurai A Efficient brain tumor detection and classification using magnetic resonance imaging Biomedical Physics & Engineering Express 2021 7 5 055007 10.1088/2057-1976/ac0ccc 34260415

[10] Gallassi R Montagna P Morreale A Lorusso S Tinuper P Neuropsychological, electroencephalogram and brain computed tomography findings in motor neuron disease European neurology 1989 29 2 115 120 10.1159/000116391 2785038

[11] Drenthen J Maathuis EM Visser GH van Doorn PA Blok JH Limb motor nerve dysfunction in Miller Fisher syndrome Journal of the Peripheral Nervous System 2013 18 1 25 29 10.1111/jns5.12003 23521640

[12] Gayathri SG Joseph Jawhar S A Novel IR analyzer based property extraction for segmented branch retinal artery occlusion and GWO-CNN based classification–an ophthalmic outcome IETE Journal of Research 2023 69 4 2164 2176 10.1080/03772063.2021.1886876

[13] Area-Gomez E Larrea D Yun T Xu Y Hupf J Lipidomics study of plasma from patients suggest that ALS and PLS are part of a continuum of motor neuron disorders Scientific Reports 2021 11 1 13562 10.1038/s41598-021-92112-3 34193885 PMC8245424

[14] Carbayo Á Borrego-Écija S Turon-Sans J Cortés-Vicente E Molina-Porcel L Clinicopathological correlates in the frontotemporal lobar degeneration–motor neuron disease spectrum Brain 2024 147 7 2357 2367 10.1093/brain/awae011 38227807 PMC11224598

[15] Kwon HS Park Y Kim JH Kim SH Jun J-B Prevalence of motor neuron diseases in gout patients: a nationwide population-based cohort study Neurological Sciences 2023 44 2 593 600 10.1007/s10072-022-06451-8 36271260

[16] Kanthavel R Dhaya R Ahilan A AI-based efficient WUGS network channel modeling and clustered cooperative communication ACM Transactions on Sensor Networks 2022 18 3 10.1145/3469034

[17] Karthikeyan M Subashini TS Srinivasan R Santhanakrishnan C Ahilan A YOLOAPPLE: augment Yolov3 deep learning algorithm for apple fruit quality detection Signal, Image and Video Processing 2024 18 1 119 128 10.1007/s11760-023-02710-z

[18] Agosta F Spinelli EG Riva N Fontana A Basaia S Survival prediction models in motor neuron disease European Journal of Neurology 2019 26 9 1143 1152 10.1111/ene.13957 30920076

[19] Lauraitis A Maskeliūnas R Damaševičius R Połap D Woźniak M A smartphone application for automated decision support in cognitive task-based evaluation of central neuron system motor disorders IEEE Journal of Biomedical and Health Informatics 2019 23 5 1865 1876 10.1109/JBHI.2019.2891729 30629520

[20] Hassanpour A Moradikia M Adeli H Khayami SR Shamsinejadbabaki P A novel end-to-end deep learning scheme for classifying multi-class motor imagery electroencephalography signals Expert Systems 2019 36 6 12494 10.1111/exsy.12494

[21] Ramakrishnan J Mavaluru D Sakthivel RS Alqahtani AS Mubarakali A Brain–computer interface for amyotrophic lateral sclerosis patients using deep learning network Neural Computing and Applications 2020 34 13439 13453 10.1007/s00521-020-05026-y

[22] Zhang K Xu G Han Z Ma K Zheng X Data augmentation for motor imagery signal classification based on a hybrid neural network Sensors 2020 20 16 4485 10.3390/s20164485 32796607 PMC7474427

[23] Greco A Chiesa MR Da Prato I Romanelli AM Dolciotti C Using blood data for the differential diagnosis and prognosis of motor neuron diseases: a new dataset for machine learning applications Scientific Reports 2021 11 1 3371 10.1038/s41598-021-82940-8 33564045 PMC7873306

[24] Subasi A Mian Qaisar S The ensemble machine learning-based classification of motor imagery tasks in brain-computer interface Journal of Healthcare Engineering 2021 10.1155/2021/1970769 PMC8595002 34795879

[25] Sekar G Sivakumar C Logeshwaran J NMLA: the smart detection of motor neuron disease and analyze the health impacts with neuro machine learning model NeuroQuantology 2022 20 8 892 899

[26] Bede P Murad A Lope J Shing SLH Finegan E Phenotypic categorisation of individual subjects with motor neuron disease based on radiological disease burden patterns: a machine-learning approach Journal of the Neurological Sciences 2022 432 120079 10.1016/j.jns.2021.120079 34875472

[27] Toh C Keslake A Payne T Onwuegbuzie A Harding J Analysis of brain and spinal MRI measures in a common domain to investigate directional neurodegeneration in motor neuron disease Journal of Neurology 2023 270 3 1682 1690 10.1007/s00415-022-11520-1 36509983 PMC9971079

[28] Sarwinda D Paradisa RH Bustamam A Anggia P Deep learning in image classification using residual network (ResNet) variants for detection of colorectal cancer Procedia Computer Science 2021 179 423 431 10.1016/j.procs.2021.01.025

[29] Aliyu HA Razak MAA Sudirman R Ramli N A deep learning AlexNet model for classification of red blood cells in sickle cell anemia International Journal of Artificial Intelligence 2020 9 2 221 228 10.11591/ijai.v9.i2.pp221-228

[30] Anand R Shanthi T Nithish MS Lakshman S Face recognition and classification using GoogleNET architecture Das K Bansal J Deep K Nagar A Pathipooranam P Naidu R Soft Computing for Problem Solving Advances in Intelligent Systems and Computing 1048 Singapore Springer 2020 261 269 10.1007/978-981-15-0035-0_20

